# Electrical storm reveals worse prognosis compared to myocardial infarction complicated by ventricular tachyarrhythmias in ICD recipients

**DOI:** 10.1007/s00380-021-01844-9

**Published:** 2021-04-26

**Authors:** Julian Müller, Michael Behnes, Tobias Schupp, Dominik Ellguth, Gabriel Taton, Linda Reiser, Niko Engelke, Martin Borggrefe, Thomas Reichelt, Armin Bollow, Ibrahim El-Battrawy, Kathrin Weidner, Seung-Hyun Kim, Christian Barth, Uzair Ansari, Dirk Große Meininghaus, Muharrem Akin, Kambis Mashayekhi, Ibrahim Akin

**Affiliations:** 1 First Department of Medicine, Faculty of Medicine Mannheim, University of Heidelberg, Theodor-Kutzer-Ufer 1-3, 68167 Mannheim, Germany; 2 Department of Cardiology, Carl-Thiem-Klinikum Cottbus, Cottbus, Germany; 3 Department of Cardiology and Angiology, Hannover Medical School, Hannover, Germany; 4 Department of Cardiology and Angiology II, University Heart Center Freiburg, Bad Krozingen, Germany

**Keywords:** Electrical storm, Acute myocardial infarction, Ventricular tachycardia, Ventricular fibrillation, Acute heart failure, Heart failure, Sudden cardiac death, MACE, Mortality, Hospitalization

## Abstract

Both acute myocardial infarction complicated by ventricular tachyarrhythmias (AMI–VTA) and electrical storm (ES) represent life-threatening clinical conditions. However, a direct comparison of both sub-groups regarding prognostic endpoints has never been investigated. All consecutive implantable cardioverter-defibrillator (ICD) recipients were included retrospectively from 2002 to 2016. Patients with ES apart from AMI (ES) were compared to patients with AMI accompanied by ventricular tachyarrhythmias (AMI–VTA). The primary endpoint was all-cause mortality at 3 years, secondary endpoints were in-hospital mortality, rehospitalization rates and major adverse cardiac event (MACE) at 3 years. A total of 198 consecutive ICD recipients were included (AMI–VTA: 56%; ST-segment elevation myocardial infarction (STEMI): 22%; non-ST-segment myocardial infarction (NSTEMI) 78%; ES: 44%). ES patients were older and had higher rates of severely reduced left ventricular ejection fraction (LVEF) < 35%. ES was associated with increased all-cause mortality at 3 years (37% vs. 19%; *p* = 0.001; hazard ratio [HR] = 2.242; 95% CI 2.291–3.894; *p* = 0.004) and with increased risk of first cardiac rehospitalization (44% vs. 12%; *p* = 0.001; HR = 4.694; 95% CI 2.498–8.823; *p* = 0.001). This worse prognosis of ES compared to AMI–VTA was still evident after multivariable adjustment (long-term all-cause mortality: HR = 2.504; 95% CI 1.093–5.739; *p* = 0.030; first cardiac rehospitalization: HR = 2.887; 95% CI 1.240–6.720; *p* = 0.014). In contrast, the rates of MACE (40% vs. 32%; *p* = 0.326) were comparable in both groups. At long-term follow-up of 3 years, ES was associated with higher rates of all-cause mortality and rehospitalization compared to patients with AMI–VTA.

## Introduction

Both, electrical storm (ES) and acute myocardial infarction complicated by ventricular tachyarrhythmias (AMI–VTA) represent life-threatening clinical conditions. Up to 6% of acute coronary syndrome (ACS) cases are complicated by ventricular tachyarrhythmias and associated with an unfavorable clinical outcome [1–3]. Irreversible myocardial ischemia alleviates the development of focal and non-focal arrhythmogenic sources degenerating into ventricular tachycardia (VT) or fibrillation (VF) [4, 5]. Hemodynamic instability due to ventricular tachyarrhythmias is associated with highest mortality. However, these patients are not well-represented in randomized controlled trials and solid data about their prognosis is scarce [6]. Nevertheless, late occurrence of ventricular tachyarrhythmias after MI is associated with worst prognosis [7].

Moreover, cardiomyopathies, myocarditis, electrolyte disorders or channelopathies are well established triggers for ventricular tachyarrhythmias [8]. ES is defined as at least three distinct episodes of sustained VT or VF requiring implantable cardioverter defibrillator (ICD) therapy within 24 h [9]. ES patients reveal a wide range of symptoms from asymptomatic delivery of anti-tachycardia pacing (ATP) to hemodynamic instability accompanied by multiple ICD-related shocks. Especially in patients with ICD and heart failure (HF), ES is a rising epidemiological problem [10]. Due to increasing rates of ICD implantation worldwide, the detection and diagnostic confirmation of ES has accelerated [11]. Nowadays, the prevalence of ES in ICD recipients is estimated around 20%, with rates of 4% in primary preventive and up to 28% in secondary preventive ICD recipients [10, 12–14]. Potential triggers of ES are new-onset or worsening HF, changes of antiarrhythmic drug therapies, diarrhea, hypokalemia and psychological stress. Furthermore, severe systolic dysfunction, chronic kidney disease and VT as the initial arrhythmia are regarded as independent and established predictors of ES [10, 14].

However, the prognostic impact of both high-risk diseases, i.e. AMI complicated by ventricular tachyarrhythmias (AMI–VTA) and of ES, has never been compared before. Therefore, the present study comparatively evaluates the long-term prognostic impact of AMI–VTA compared to ES independent from AMI in consecutive ICD recipients.

## Methods

### Study population

**Fig. 1:**
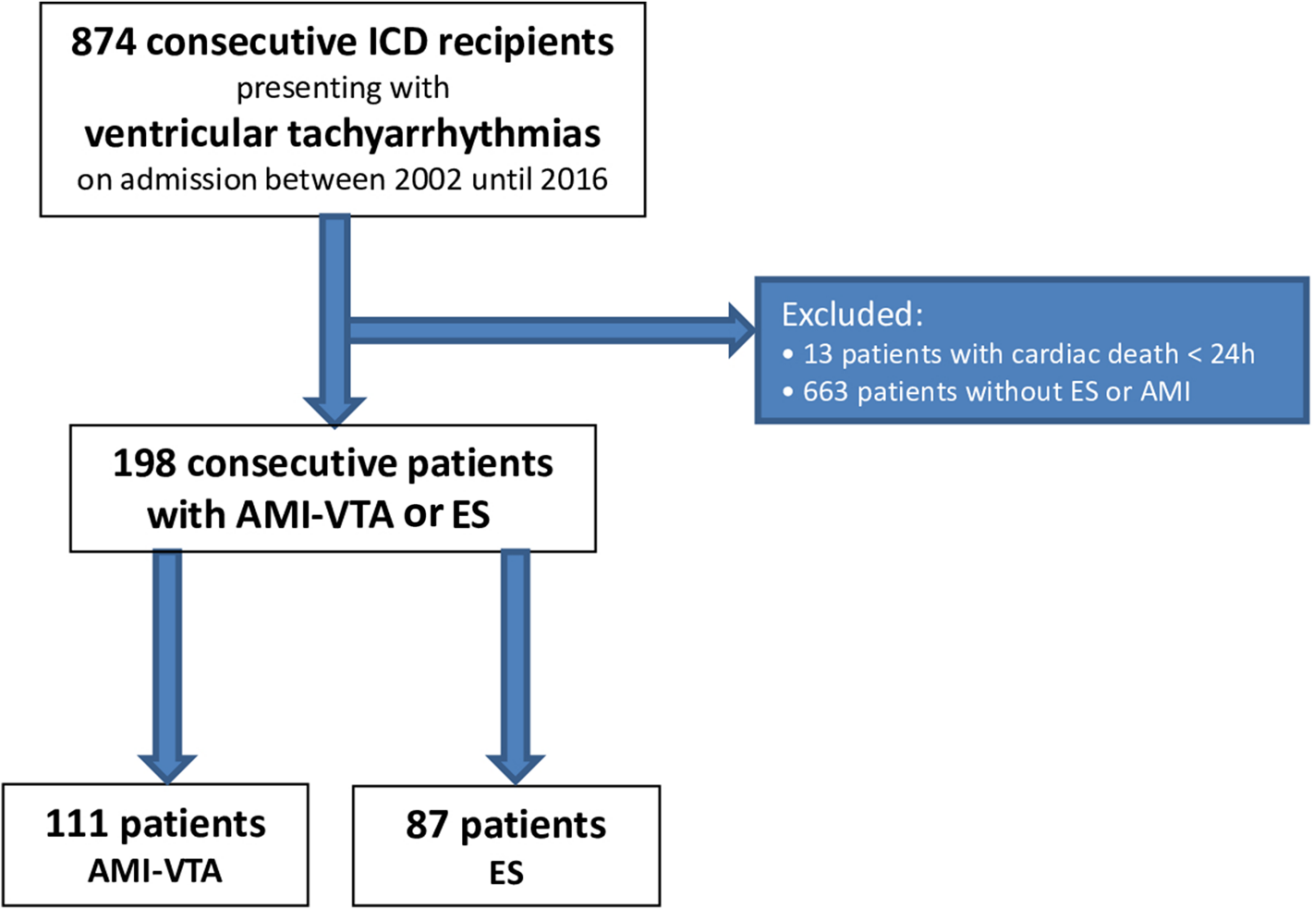
Flow chart of the study population

The present study included all consecutive ICD recipients presenting with AMI–VTA and those with ES apart from AMI presenting from 2002 until 2016 at one institution (Fig. 1).

Ventricular tachyarrhythmias comprised VT and VF, as defined by current international guidelines [6, 8]. VT was classified in the presence of a regular RR interval, large QRS morphology, changing polarity of QRS deflections during tachycardia compared to sinus rhythm and sudden onset of tachycardia. A heart rate > 250/min with irregular RR intervals classified VF [6, 8]. Ventricular tachyarrhythmias were documented by ICD and in some cases additionally by 12-lead electrocardiogram (ECG), ECG tele-monitoring, or in case of unstable course or during resuscitation by external defibrillator monitoring. Left ventricular ejection fraction (LVEF) was measured prior to discharge by standardized transthoracic echocardiogram after stabilization of ES. To a small extent and only if available, LVEF was assessed bedside on admission or during intensive care in hemodynamically unstable patients with expected death within the acute phase of AMI–VTA or ES already.

All relevant clinical data was documented using the electronic hospital information system, ICD protocols, discharge letters, patients’ files, 12-lead ECGs and Holter-ECGs being assessed during clinical routine. In detail, data documentation comprised baseline characteristics, prior medical history, prior medical treatment, length of index stay, all findings of laboratory values at baseline, data derived from all non-invasive or invasive cardiac diagnostics and device therapies, such as coronary angiography, electrophysiological examination, as well as imaging modalities, such as echocardiography or cardiac magnetic resonance imaging (cMRI). Documentation period lasted from index event until 2016. Independent cardiologists performed documentation of all medical data blinded to final data analyses.

The present study is derived from an analysis of the “Registry of Malignant Arrhythmias and Sudden Cardiac Death—Influence of Diagnostics and Interventions (RACE-IT)” and represents a single-center registry including consecutive patients presenting with ventricular tachyarrhythmias and aborted cardiac arrest (SCD) being acutely admitted to the University Medical Center Mannheim (UMM), Germany (clinicaltrials.gov identifier: NCT02982473) from 2002 until 2016. The registry was carried out according to the principles of the declaration of Helsinki and was approved by the medical ethics committee II of the Faculty of Medicine Mannheim, University of Heidelberg, Germany.

### Definition of study groups, inclusion and exclusion criteria

Patients with AMI–VTA were compared to patients with ES apart from AMI. All patients already had an activated ICD prior to inclusion.

The AMI–VTA group comprised all consecutive ICD recipients presenting with AMI complicated by ventricular tachyarrhythmias during inclusion period. AMI–VTA patients with cardiac death < 24 h were excluded. AMI was defined according to European guidelines [8, 15–18]. ST-segment-elevation myocardial infarction (STEMI) was defined as a novel rise in the ST segment in two adjacent ECG leads, greater than 2 mm (0.2 mV) for males and greater than 1.5 mm (0.15 mV) in females in all leads except for V2 and V3, where it must be greater than 1 mm (0.1 mV). Additional ECG criteria were new ST depression or inversion, T wave alterations, Q waves or new left bundle branch block. Non-ST-segment-elevation myocardial infarction (NSTEMI) was defined as the presence of an ACS with a troponin I increase of above the 99th percentile of a healthy reference population. The culprit lesion was defined as an acute complete thrombotic occlusion for STEMI and any relevant critical coronary stenosis for NSTEMI with the potential need for coronary revascularization either by PCI or coronary artery bypass grafting. Target vessel revascularization (TVR) was defined as any percutaneous intervention of the target lesion or bypass surgery of the target vessel performed for stenosis or other complication of the target lesion. The presence of a coronary culprit lesion with the potential need for coronary revascularization either by PCI or CABG was mandatory for both diagnoses of NSTEMI and STEMI. Evidence of regional wall motion abnormalities was also included in AMI diagnosis as far as available.

The ES group comprised all ICD recipients with ES during inclusion period. ES patients with cardiac death < 24 h were excluded. ES was defined as ≥ 3 episodes of ventricular tachyarrhythmias delimited by at least 5 min leading to appropriate ICD therapy during a single time period of 24 h.

Patients with cardiac death at 24 h were excluded. Cardiac death was defined as cardiac death occurring within 24 h after onset of ventricular tachyarrhythmias (VT or VF) or ES. Furthermore, patients with cardiac death within 24 h of an assumed unstable cardiac condition leading to cardiac death, such as high degree AV-block, asystole, acute heart failure (AHF), cardiogenic shock or cardiopulmonary resuscitation (CPR) even in the absence of ventricular tachyarrhythmias, were excluded [8]. Each patient was counted only once for inclusion.

### Definition of endpoints

The primary endpoint was all-cause mortality at three years. The observational period for primary and secondary endpoints started with the index presentation at our medical center with ES or AMI-VTA and lasted for 3 years thereafter. Secondary endpoints comprised in-hospital mortality, first rehospitalization and major adverse cardiac events (MACE) both at three years. First rehospitalization comprised first rehospitalization due to VT, VF, CPR, AHF, AMI, inappropriate ICD shock or stroke at 3 years of follow-up. MACE were defined as the composite of AMI, target vessel revascularization (TVR) by percutaneous coronary intervention (PCI) or coronary artery bypass grafting (CABG) and all-cause mortality [19].

All-cause mortality was documented using our electronic hospital information system and by directly contacting state resident registration offices (“bureau of mortality statistics”) across Germany. Identification of patients was verified by name, surname, date of birth and registered living address.

### Statistical methods

Quantitative data is presented as mean ± standard error of mean (SEM), median and interquartile range (IQR) and ranges depending on the distribution of the data. It was compared using the student’s *t* test for normally distributed data or the Mann–Whitney *U* test for non-parametric data. Deviation from a Gaussian distribution were tested by the Kolmogorov–Smirnov test. Spearman’s rank correlation for non-parametric data was used to test univariate correlations. Qualitative data are presented as absolute and relative frequencies and compared using the Chi^2^ test or the Fisher’s exact test, as appropriate.

The following analyses were applied stepwise to evaluate the prognostic value of predefined variables for all-cause mortality: Kaplan–Meier survival curves were calculated with log-rank testing for statistical significance. Uni-variable hazard ratios (HR) are given together with 95% confidence intervals. Multivariable Cox regression models with all-cause mortality and rehospitalization as the dependent variable were developed using the “forward selection” option. Multivariable models were adjusted both by univariably statistically significant variables such as treatment with statin or amiodarone and impaired LVEF (*p* < 0.05), as well as with clinically relevant variables such as age, diabetes, chronic kidney disease (CKD) and coronary artery disease (CAD).

The result of a statistical test was considered significant for *p* < 0.050, and a statistical trend was defined as *p* < 0.100. SPSS (Version 25, IBM Armonk, New York, USA) was used for statistics.

## Results

### Study population

A total of 198 consecutive ICD recipients were included, of which 44% suffered from at least one episode of ES and 56% from AMI–VTA (Fig. 1).

As illustrated in Table 1, most patients were males (at least 82%). ES patients were older (70 years vs. 66 years; *p* = 0.026); whereas AMI–VTA patients presented with significantly higher rates of smoking (38% vs. 17%; *p* = 0.001) and cardiac family history (20% vs. 8%; *p* = 0.020). Other cardiovascular risk factors were comparable between both groups. However, AMI–VTA patients suffered from cardiogenic shock (20% vs. 2%; *p* = 0.001) and underwent CPR (52% vs. 6%; *p* = 0.001) more often. In contrast, dilated cardiomyopathy (DCM) (16% vs. 0%; *p* = 0.001) was more common in ES patients. In addition, ablation therapy due to VT was more often performed in ES patients (21% vs. 5%; *p* = 0.001).

**Table 1:** Baseline characteristics of AMI–VTA and ES

Characteristic	AMI-VTA^a^ (*n* = 111; 56%)		ES^a^ (*n* = 87; 44%)		*p* value
Age, median (range)	66 (40–79)		70 (22–85)		**0.026**
Males, *n* (%)	91 (82)		74 (85)		0.564
Cardiovascular risk factors, *n* (%)					
Arterial hypertension	76 (69)		55 (63)		0.438
Diabetes mellitus	35 (32)		22 (25)		0.335
Hyperlipidemia	45 (41)		37 (43)		0.778
Smoking	42 (38)		15 (17)		**0.001**
Cardiac family history	22 (20)		7 (8)		**0.020**
Comorbidities, *n* (%)					
Cardiogenic shock	22 (20)		2 (2)		**0.001**
CPR	58 (52)		5 (6)		**0.001**
Atrial fibrillation	38 (34)		36 (41)		0.302
Cardiomyopathy	0 (0)		21 (24)		**0.001**
Dilated cardiomyopathy	0 (0)		14 (16)		**0.001**
Hypertrophic cardiomyopahty	0 (0)		2 (2)		1.000
HNOCM	0 (0)		1 (1)		1.000
ARVD	0 (0)		1 (1)		1.000
Cardiac sarcoidosis	0 (0)		1 (1)		1.000
Long QT syndrome	0 (0)		1 (1)		1.000
Brugada syndrome	0 (0)		1 (1)		1.000
Electrolyte disorders	0 (0)		2 (2)		1.000
Electrophysiological examination, *n* (%)	20 (18)		21 (24)		0.292
VT ablation	5 (5)		18 (21)		**0.001**
Laboratory data, (mean ± SEM)					
Hemoglobin [g/dl]	13.0 ± 0.2		13.2 ± 0.2		0.670
Potassium [mmol/l]	4.1 ± 0.1		4.0 ± 0.1		0.422
Creatinine [mg/dl]	1.4 ± 0.1		1.4 ± 0.1		0.590
Medication at discharge, *n* (%)					
Beta-blocker	99 (94)		80 (95)		0.589
ACE-inhibitor/ ARB	93 (88)		67 (80)		0.134
Statin	98 (93)		50 (60)		**0.001**
Amiodarone	21 (20)		45 (54)		**0.001**
ECG data, (mean ± SEM)					
PQ	179 ± 8		220 ± 11		**0.005**
QRS	117 ± 7		127 ± 13		0.414
QT	410 ± 16		440 ± 14		**0.033**
LVEF, *n* (%)					
≥ 55%	14 (13)		9 (10)		**0.008**
54–45	13 (12)		7 (8)		
44–35%	27 (24)		9 (10)		
< 35%	43 (38)		55 (64)		
Not documented	14 (13)		7 (8)		
Type of ICD, *n*(%)					
ICD	99 (89)		76 (87)		0.878
CRT-D	8 (7)		8 (9)		
s-ICD	4 (4)		3 (3)		
ICD indication, *n*(%)					
Primary prevention	39 (35)		32 (37)		0.764
Secondary prevention	72 (65)		54 (63)		
ICD programming, bpm, median (IQR)					
VT detection threshold	171 (167–181)		171 (159–176)		0.084
VF detection threshold	214 (214–222)		214 (214–222)		0.925

*AMI* acute myocardial infarction, *ACE* angiotensin-converting enzyme, *AMI* acute myocardial infarction, ARB, angiotensin receptor blocker, ARVD arrhythmogenic right ventricular dysplasia, CPR cardiopulmonary resuscitation, *CRT-D* cardiac resynchronisation therapy defibrillator, *ECG* electrocardiogram, *ES* electrical storming, *HNOCM* hypertrophic non-obstructive cardiomyopathy, *ICD* implantable cardioverter-defibrillator, *IQR* inter quartile range, *LVEF* left ventricular ejection fraction, *SEM* standard error of measurement, *VTA* ventricular tachyarrhythmias, *VF* ventricular fibrillation, *VT* ventricular tachycardia

^a^All patients have an ICD

Bold values indicate statistical significance (*p* < 0.05)

AMI–VTA patients were treated more often with statins (93% vs. 60%; *p* = 0.001), ES patients more often treated with amiodarone (54% vs. 20%; *p* = 0.001). The ECG recorded at baseline showed significantly longer PQ (220 ms ± 11 ms vs. 179 ms ± 8 ms; *p* = 0.005) and QT intervals (440 ms ± 14 ms vs. 410 ms ± 16 ms, *p* = 0.033) in ES patients. In addition, ES patients suffered more often from severely reduced LVEF (69% vs. 44%; *p* = 0.008) (Table 1).

The most common device was a conventional ICD (89% vs. 87%), followed by transvenous CRT-D (7 vs. 9%) and subcutaneous ICD (4% vs. 3%). Those devices were mostly implanted for secondary prevention (65% vs. 63%) (Table 1).

### Distribution of CAD and VTA in both groups

Within AMI–VTA patients, STEMI was less common than NSTEMI (22% vs. 78%). AMI was more often complicated by VF than VT (52% vs. 48%) and infarct-related VT occurred three times more often < 48 h than > 48 h post AMI (34% vs. 14%). AMI–VTA patients underwent coronary angiography more often (88% vs. 52%; *p* = 0.001). CAD was significantly more common in AMI–VTA patients (87% vs. 44%; *p* = 0.001). Coronary single vessel disease was more frequent in AMI–VTA patients (28% vs. 5%; *p* = 0.009); whereas, CABG was more common in ES patients (24% vs. 10%; *p* = 0.011). Furthermore, an intracoronary thrombus was more often found in AMI–VTA patients (7% vs. 0%; *p* = 0.008) (Table 2).

**Table 2:** Characteristics of coronary artery disease in between AMI–VTA and ES

	AMI–VTA^a^ (*n* = 111; 56%)		ES^a^ (*n* = 87; 44%)		*p* value
STEMI	24	(22)	0	(0)	**0.001**
NSTEMI	87	(78)	0	(0)	**0.001**
With VT	53	(48)	–	–	–
VT < 48 h	38	(34)	–	–	–
VT > 48 h	15	(14)	–	–	–
With VF	58	(52)	–	–	–
Coronary angiography, overall	98	(88)	45	(52)	**0.001**
Coronary artery disease	96	(87)	38	(44)	**0.001**
No evidence of CAD	2	(2)	7	(8)	**0.001**
1-vessel	28	(28)	4	(5)	**0.009**
2-vessel	32	(33)	10	(11)	0.239
3-vessel	36	(37)	24	(28)	0.070
Prior CABG	11	(10)	21	(24)	**0.011**
Intracoronary thrombus	8	(7)	0	(0)	**0.008**
CPR during coronary angiography	6	(5)	0	(0)	0.069
PCI, *n* (%)	76	(69)	14	(16)	**0.001**
Target vessel revascularization, *n* (%)					
RCA	26	(23)	4	(5)	**0.001**
LMT	0	(0)	4	(5)	**0.036**
LAD	46	(41)	7	(8)	**0.001**
LCx	20	(18)	6	(7)	**0.033**
RIM	0	(0)	0	(0)	–
Bypass graft	0	(0)	0	(0)	–
Sent to CABG	0	(0)	0	(0)	–

*AMI* acute myocardial infarction, *CABG* coronary artery bypass graft, *CAD* coronary artery disease, *ES* electrical storming, *ICD* implantable cardioverter-defibrillator, *LAD* left anterior descending, *LCx* left circumflex, *LMT* left main trunk, *NSTEMI* non-ST-segment-elevation myocardial infarction, *PCI* percutaneous coronary intervention, *RCA* right coronary artery, *RIM* ramus intermedius, *STEMI* ST-segment-elevation myocardial infarction, *TVR* target vessel revascularization, *VF* ventricular fibrillation, *VT* ventricular tachycardia, *VTA* ventricular tachyarrhythmia

^a^All patients had an ICD

Bold values indicate statistical significance (*p* < 0.05)

AMI–VTA patients had higher rates of PCI during index coronary angiography (69% vs. 16%; *p* = 0.001). Among AMI–VTA patients, TVR was more often performed in the right coronary artery (23% vs. 5%; 0.001), left anterior descending artery (41% vs. 8%; *p* = 0.001) and left circumflex artery (18% vs. 7%; *p* = 0.033); whereas, ES patients had TVR more often at the left main trunk (5% vs. 0%; *p* = 0.036) (Table 2).

### Primary endpoint of long-term all-cause mortality

**Fig. 2:**
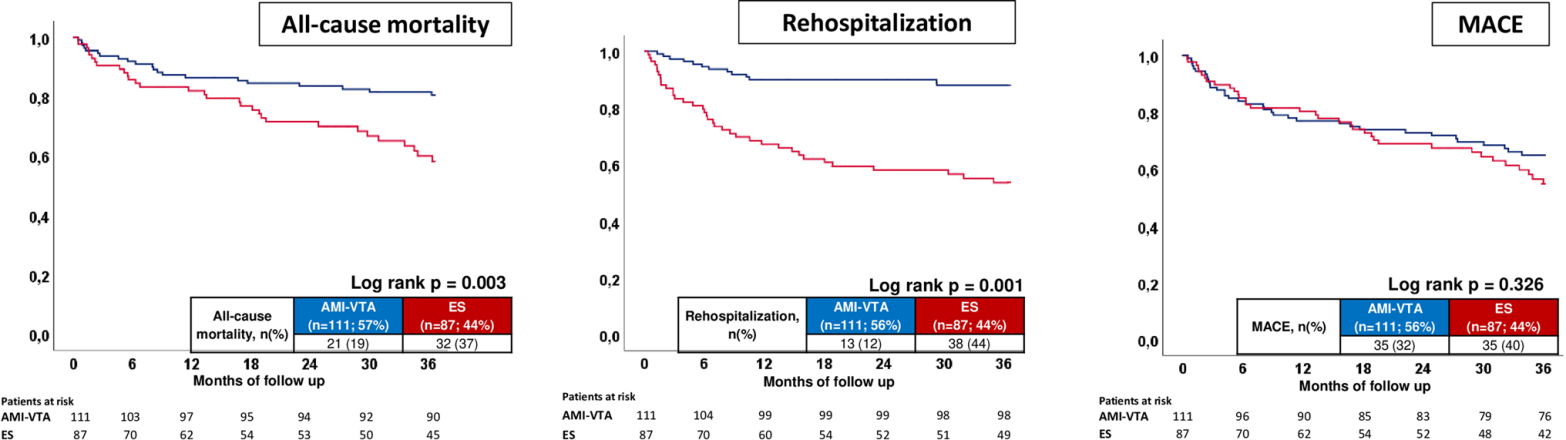
Prognostic impact of acute myocardial infarction complicated by ventricular tachyarrhythmias (AMI–VTA) compared to electrical storm (ES) on long-term all-cause mortality (left panel), overall rehospitalization (middle panel) and major adverse cardiac events (MACE) (right panel) in implantable cardioverter-defibrillator (ICD) recipients

At three years of long-term follow-up (mean 35 months) starting with index event of ES or AMI–VTA at presentation in our medical center, ES was associated with higher rates of all-cause mortality compared to AMI-VTA patients (37% vs. 19%, log rank *p* = 0.003; Table 3; Fig. 2, left panel), corresponding to a 2.2-fold increased risk of all-cause death (HR 2.242; 95% CI 1.291–3.894, *p* = 0.004). Consistently, all-cause mortality of ES patients remained even higher when only ES patients with ischemic heart disease were compared with AMI–VTA patients (49% vs. 19%, log rank *p* = 0.014, data not shown).

**Table 3:** Primary and secondary endpoints

Characteristics	AMI–VTA (*n* = 111; 56%)		ES (*n* = 87; 44%)		*p* value
Primary endpoint, *n* (%)					
All-cause mortality	21	(19)	32	(37)	**0.005**
Secondary endpoints, *n* (%)					
In-hospital mortality	5	(5)	2	(2)	0.404
First rehospitalization					
Overall	13	(12)	38	(44)	**0.001**
VT	0	(0)	19	(23)	**0.001**
VF	1	(0.9)	0	(0)	1.000
CPR	0	(0)	2	(2)	1.000
Acute heart failure	6	(6)	15	(17)	**0.010**
Acute myocardial infarction	2	(2)	1	(1)	1.000
Inappropriate ICD shock	4	(4)	0	(0)	1.000
Stroke	0	(0)	1	(1)	1.000
MACE	35	(32)	35	(40)	0.204
All-cause mortality	21	(19)	32	(37)	**0.005**
AMI	4	(4)	1	(1)	0.275
TVR	15	(14)	2	(2)	**0.005**
CABG	–	–	–	–	–

*AMI* acute myocardial infraction, *CABG* coronary artery bypass graft, *CPR* cardiopulmonary resuscitation, *ES* electrical storming, *ICD* implantable cardioverter-defibrillator, *MACE* major adverse cardiac events, *TVR* target vessel revascularization, *VF* ventricular fibrillation, *VT* ventricular tachycardia, *VTA* ventricular tachyarrhythmia

Bold values indicate statistical significance (*p* < 0.05)

Furthermore, ES patients still remained at highest risk for mortality when AMI patients with occurrence of ventricular tachyarrhythmias < 48 h after AMI were excluded from the AMI–VTA group within this analysis (37% vs. 25%, log rank *p* = 0.039, data not shown).

### Secondary endpoints

No differences were observed regarding in-hospital mortality in between ES and AMI–VTA patients (2% vs. 5%; *p* = 0.404). In contrast, ES patients revealed significantly higher rates of overall first cardiac rehospitalization (44% vs. 12%; log rank *p* = 0.001), mainly attributed to VT (23% vs. 0%; *p* = 0.001) and AHF (17% vs. 6%; *p* = 0.010). The risk of cardiac rehospitalization for ES patients was 4.7 times higher compared to AMI–VTA patients (HR 4.694, 95% CI 2.498–8.823; *p* = 0.001; Table 3; Fig. 2, middle panel).

Notably, rates of MACE were equally distributed between ES and AMI–VTA patients (40% vs. 32%; *p* = 0.204; Table 3; Fig. 2, right panel). For most patients all-cause mortality was the reason for MACE. Apart from that, TVR was significantly more often performed in AMI-VTA patients during follow-up time. Of note, 23 patients with ES developed recurrences of ES within follow-up (26%).

### Multivariable Cox models

Increased long-term all-cause mortality in ES patients was still evident after multivariable adjustment (HR 2.504; 95% CI 1.093–5.739; *p* = 0.030), besides age (HR 1.042; 95% CI 1.001–1.085; *p* = 0.043) and CKD (HR 6.876; 95% CI 2.602–18.170; *p* = 0.001) (Table 4). Furthermore, ES was still associated with 2.9-fold increased risk of rehospitalization (HR 2.887; 95% CI 1.240–6.720; *p* = 0.014) (Table 5).

**Table 4:** Multivariable Cox regression for long-term all-cause mortality at 2.5 years

Variable	HR	95% CI	*p* value
Age	1.042	1.001–1.085	**0.043**
Diabetes	1.235	0.597–2.555	0.569
Chronic kidney disease	6.876	2.602–18.170	**0.001**
CAD	1.629	0.607–4.370	0.333
Amiodarone treatment	1.598	0.782–3.265	0.198
LVEF ≤ 35%	1.180	0.753–1.850	0.469
Statin treatment	0.797	0.328–1.935	0.616
Electrical storm	2.504	1.093–5.739	**0.030**

*CAD* coronary artery disease, *CI* confidence interval, *HR* hazard ratio, *LVEF* left ventricular ejection fraction; Chronic kidney disease defines as creatinine > 1.2 mg/dl

Bold values indicate statistical significance (*p* < 0.05)

**Table 5:** Multivariable Cox regression for long-term rehospitalization at 2.5 years

Variable	HR	95% CI	*p* value
Age	1.006	0.980–1.032	0.667
Diabetes	0.808	0.390–1.671	0.564
Chronic kidney disease	0.736	0.387–1.399	0.349
CAD	0.692	0.312–1.532	0.363
Amiodarone treatment	1.653	0.812–3.365	0.165
LVEF ≤ 35%	1.054	0.753–1.476	0.757
Statin treatment	0.593	0.285–1.231	0.161
Electrical storm	2.887	1.240–6.720	**0.014**

*CAD* coronary artery disease, *CI* confidence interval, *HR* hazard ratio, *LVEF* left ventricular ejection fraction; Chronic kidney disease defines as creatinine > 1.2 mg/dl

Bold values indicate statistical significance (*p* < 0.05)

## Discussion

The present study evaluates the prognostic impact of AMI complicated by ventricular tachyarrhythmias compared to that of ES apart from AMI on long-term all-cause mortality, as well as rates of rehospitalization and MACE in consecutive ICD recipients. This data suggests that ES is associated with worse long-term prognosis, even when compared to high-risk patients with AMI–VTA and even in the presence of an activated ICD. ES patients were associated with increased all-cause mortality and rehospitalization rates at 3 years, mainly attributed to recurrent VT and AHF. Worse prognosis in ES patients was still evident after multivariable adjustment.

Despite improvements in device therapies, SCD still occurs in 2% of all ICD patients. Furthermore, the number of recurrences of VT and VF is directly related to death rates [20]. A recent meta-analysis comparing ES patients to patients without any history of ventricular tachyarrhythmias revealed a 3.1-fold increased risk of all-cause mortality among ES patients. Interestingly, increasing rates of mortality were still evident between ES patients and patients with a history of ventricular tachyarrhythmias apart from ES [21]. However, this meta-analysis included all potential comorbidities triggering ventricular tachyarrhythmias and focused only on the risk of all-cause mortality and the composite endpoint of all-cause mortality, heart transplantation and hospitalization due to HF. In contrast, the present study explicitly included all patients with AMI–VTA. The adverse prognostic impact of ES compared to AMI–VTA was still evident despite the presence of an activated ICD.

Up to 6% of patients with AMI develop VT or VF during the first 48 h after onset of ischemia-related symptoms, commonly prior to or during coronary revascularization. Due to better revascularization strategies and preventive measures for CAD the incidence of AMI–VTA has declined in recent decades [8].

Acute myocardial ischemia favors electrical instability provoking ventricular tachyarrhythmias in patients with AMI. During the acute phase of ischemia a multifactorial sequence of events results in ionic imbalance, less reduced resting membrane potential and reduced conduction velocity [22]. One important contributor in arrhythmogenesis is the intracellular Ca^2+^ overload resulting in spontaneous Ca^2+^ oscillations triggering early and delayed after-depolarizations induced ectopic beats [23]. Whereas, basic macro re-entry mechanisms from heterogenous substrates might pose the responsible mechanism for ventricular tachyarrhythmias during ischemia, impulse initiation caused by after-depolarizations appears the dominant mechanism in reperfusion [24].

Within the present study, half of all AMI patients underwent CPR related to ventricular tachyarrhythmias reflecting hemodynamic instability and cardiogenic shock. In contrast, increased antiarrhythmic therapies such as VT ablation and amiodarone treatment were present in patients with ES. Nevertheless, ES patients died significantly more often during follow-up time compared to patients with AMI–VTA. These findings suggest that ES patients, even when compared to a highest-risk cohort of patients with AMI–VTA represent a population of utmost risk for cardiovascular death. Even after exclusion of patients suffering from VTA within 48 h after AMI ES patients remained at highest for subsequent mortality [3]. Accordingly, this impaired prognosis could be also confirmed in multivariable regression models.

Several pathophysiological hypotheses explaining the increased risk of death among ES patients are available. Some authors revealed an increased mortality rate among patients with ventricular tachyarrhythmias terminated by shocks compared to patients treated only by ATP with or without antiarrhythmic therapy [25]. Recurrent shocks might contribute to transient systolic dysfunction and AHF in terms of cardiac decompensation [26]. Another possible explanation might be that recurrent VT, especially incessant VTs, promote LV dysfunction leading to advanced HF, cardiogenic shock and death [27]. Accordingly, the results of the present study provide further understanding and affirm the hypothesis that ES might affect mortality via LV dysfunction. ES patients presented with more severely decreased LVEF and higher rates of VT recurrences. Presumably, most ES patients died from advanced ischemic heart failure with concomitant CKD. Advanced heart failure per se is a major risk factor for subsequent mortality. In addition, cardiorenal syndrome represents worst prognosis for patients presenting with ventricular tachyarrhythmias [28, 29].

Interestingly, in patients with drug-resistant ES, VT ablation provided increased survival rates. Reduction of tachyarrhythmic episodes might prevent associated decline of LVEF. And severely reduced LVEF is considered as the strongest predictor of mortality among ES patients [30, 31].

ES is also associated with increasing rates of rehospitalization alongside with each ICD shock delivery. Baensch et al. revealed a 50% risk for rehospitalization in patients with ≤ 3 shocks; whereas, those with more than 3 shocks were hospitalized 100% of the time [27]. The increased overall rehospitalization rates within this study were specifically related to VT and AHF among ES patients. This observation is in line with recent findings of Guerra et al. proposing ES as a clinical manifestation of worsening HF [21]. Also, the association between increased rehospitalization rates attributed to VT and ES was not unexpected. Monomorphic VT represents often a scar-related re-entry mechanism in the presence of heart failure due to ischemic or structural heart diseases, which can promote and sustain ES in many cases [10]. Occurrence of VT in the setting of heart failure is dependent on systolic dysfunction and within this study ES patients suffered mostly from severely reduced LVEF.

This study demonstrated the adverse prognostic impact of ES compared to AMI-VTA on long-term all-cause mortality and cardiac rehospitalization rates. Therefore, ES patients constitute a population associated with the highest risk of death. Due to this significant prognostic impact, ES patients require close clinical follow-ups and optimal pharmacological HF treatment with beta-blockers and amiodarone, as well as ICD treatment. Furthermore, since the majority of ES originate from a basic re-entry mechanism, catheter ablation is a curative approach to interrupt episodes of ventricular tachycardias. Additionally, ablation was shown to be able to decrease ES burden [30]. Notably, in some cases thoracic epidural anesthesia, renal sympathetic denervation and left cardiac sympathetic denervation are further potential therapeutic options, recently being associated with a decrease in arrhythmic burden [32–34].

In summary, ES represents a pathology with a worse prognosis compared to high-risk patients with AMI–VTA, even in the presence of an activated ICD. Further efforts should be made to unify diagnostic and therapeutic strategies for ES patients, such as invasive VT ablation and coronary angiography. Future prospective multi-center studies are needed to verify further the prognostic impact of CAD and ablation therapy both in AMI–VTA and ES patients.

## Study limitations

The main limitation of this study is the retrospective study design and the rather small sample size. Only all-cause mortality rates were documented, detailed analyses about definite cause of death were beyond the scope of the study. Rehospitalization rates were only documented within our own institution. Patients with prolonged hemodynamic instability and lethal outcome before admission and those not surviving out of hospital CPR without transfer to the heart center were not included in this study. Ablation rates among ES patients were rather low, possibly preventing to show the beneficial effect of ablation.

## Conclusions

ES is associated with higher long-term all-cause mortality and rehospitalization rates compared to AMI–VTA in ICD recipients at 3 years.
